# ERCC1 and BRCA1 mRNA expression predicts the clinical outcome of non-small cell lung cancer receiving platinum-based chemotherapy

**DOI:** 10.12669/pjms.303.4187

**Published:** 2014

**Authors:** Feng Xian-jun, Qin Xiu-guang, Zang Li, Feng Hui, Wang Wan-ling, Liu Dong, Li Ping-fa

**Affiliations:** 1 Feng Xian-jun, Department of Respiratory Medicine, The First Affiliated Hospital of Xinxiang Medical University, Weihui, China; 2 Qin Xiu-guang, Department of Thoracic Surgery, The First Affiliated Hospital of Xinxiang Medical University, Weihui, China; 3 Zang Li, Combine Traditional Chinese and Western Medicine Dept., The First Affiliated Hospital of Xinxiang Medical University, Weihui, China; 4 Feng Hui, Combine Traditional Chinese and Western Medicine Dept., The First Affiliated Hospital of Xinxiang Medical University, Weihui, China; 5 Wang Wan-ling, Department of Hematology, The First Affiliated Hospital of Xinxiang Medical University, Weihui, China; 6 Liu Dong, Department of Dermatology, The First Affiliated Hospital of Xinxiang Medical University, Weihui, China; 7 Li Ping-fa, Inspection Department, Xinxiang Medical University, Xinxiang, China

**Keywords:** Non-small cell lung cancer, ERCC1, BRCA1, RRM1, Survival time

## Abstract

***Objective:*** We conducted a perspective study to investigate the association between mRNA expression quantities of ERCC1, BRCA1, RRM1 and RRM2 and response to chemotherapy and clinical outcome of advance Non-Small Cell Lung Cancer.(NSCLC).

***Methods:*** Two hundred eight patients who were diagnosed as advanced stage NSCLC were included in our study. A fluorescence-based and real-time detection method was used to determine the relative cDNA quantification for ERCC1, BRCA1, RRM1 and RRM2, and β-actin was used as the reference gene.

***Results:*** The median expression levels of ERCC1, BRCA1, RRM1 and RRM2 mRNA were 0.67±0.17, 0.095±0.012, 0.24±0.17 and 2.45±0.32, respectively. Our study found that the low ERCC1 (OR=1.82, 95% CI=1.01-3.20) and Low BRCA1 (OR=2.53, 95%CI=1.38-4.64) mRNA expression was more likely to response to chemotherapy when compared with high expression, respectively. Multivariate Cox regression analysis indicated that patients with low mRNA expression of ERCC1 and BRCA1 attained 0.43 (OR=0.43, 95%CI=0.27-0.89) and 0.37 (OR=0.37, 95%CI=0.22-0.66) fold risk of death from NSCLC. However, we found RMM1 and RRM2 mRNA expression could not influence the response to chemotherapy and clinical outcome of NSCLC.

***Conclusion: ***ERCC1 and BRCA1 mRNA expression could be important predictive markers for individualized platinum-based chemotherapy for NSCLC patients.

## INTRODUCTION

Lung cancer remains the most common cause of mortality from malignant disease in the world for several decades, and it has been the most common cancer in China.^1^ It is estimated that there are 1.8 million new cases in 2012, 58% of them occur in the less developed countries.^1^ Non-small cell lung cancer (NSCLC) accounts for about 80% of lung cancer cases, and has a overall five-year survival rate of less than 15%.^2^^,^^3^ Most of the NSCLC patients show locally advanced or metastatic disease when diagnosed.^2 ^Platinum agents have been used as the first-line chemotherapeutic regimens to improve the clinical outcome of advanced NSCLC.^3^ Despite advances in diagnostics, surgery and chemotherapy, 70% of NSCLC patients still show metastatic disease after receiving chemotherapy.^4^ Moreover, even patients with similar clinical characteristics present different response to chemotherapy, which shows that some molecular biomarkers have a role in altering the efficacy of chemotherapy for advanced NSCLC patients. Therefore, detection of molecular markers could help design individualized chemotherapy to improve the survival of advanced NSCLC. 

Previous study reported that bulky DNA adducts by cisplatin or carboplatin are mainly repaired by nucleotide excision repair pathway.^5^ The DNA repair mechanism can allow cancer cell to repair the DNA damages caused by platinum compounds, and it can influence the anticancer effect of these agents.^6^ Excision repair cross complementing 1 (ERCC1) and breast cancer susceptibility gene 1 (BRCA1) are two key factors involved in nuclear excision repair, and increased clinical data have showed that expression.^7^^,^^8^ Ribonucleotide reductase subunit M1(RRM1) and Ribonucleotide reductase subunit M2(RRM2) are encoded by different genes on separate chromosomes and their mRNAs are differentially expressed through the cell cycle, and over-expression of RRM1 and RRM2 is correlated with resistance to chemotherapy.^9^^,^^10^

Previous studies have reported the association between ERCC1, BRCA1, RRM1 and RRM2 and NSCLC prognosis.^9^^,^^11^^-^^13^ However, the results are inconsistent.^9^^,^^11^^-^^13^ Therefore, we conducted this prospective study to investigate the role of mRNA expression quantities of ERCC1, BRCA1, RRM1 and RRM2 in NSCLC patients, and investigate their association with response to chemotherapy and clinical outcome of advanced NSCLC.

## METHODS

***Subjects: ***236 eligible patients who were diagnosed as advanced stage NSCLC were enrolled at the First Affiliated Hospital of Xinxiang Medical University between January 2009 and January 2010. Finally, 208 patients agreed to participate into our study, with participation rate of 88.1%. Excluded criteria were patients who previously received radiotherapy or chemotherapy, and those who had symptomatic brain metastases, spinal cord compression and uncontrolled massive pleural effusion. Informed consent was obtained from all patients before conducting the study. All the patients were followed up until January 2012. The study protocol was approved by the ethics committee of the First Affiliated Hospital of Xinxiang Medical University.

***Study design: ***All patients were treated with platinum-based doublets chemotherapy. The treatment regimens included 25 mg/ m^2 ^vinorelbine on day one and day eight, or 1000mg/m^2^ gemcitabine plus 75 mg/ m^2 ^cisplatin or carboplatin on day one. The chemotherapy treatment was conducted every three weeks, and then the toxicities were evaluated after chemotherapy. The chemotherapy treatment was conducted for a maximum of six cycles. When patients showed grade three or four drug-related toxcities, the dose of cytotoxic agents were immediately reduced by 25%. The response to platinum-based doublets chemotherapy was assessed by the WHO criteria.^14^

Complete remission (CR) and partial remission (PR) were defined as responsive and stable disease (SD) and progressive disease (PD) were defined as non-responsive. Overall survival (OS) was calculated from the time of diagnosis to the time of death or the end of follow-up.

***RNA isolation and cDNA quantification:*** 5 mlL whole blood samples were collected from each patient, and stored at −20C until use. For genotype determination, extraction of RNA from a peripheral blood sample was conducted by an EZNA Blood RNA Mini Kit (Omega, Berkeley, CA, US). A fluorescence-based and real-time detection method was used to determine the relative cDNA quantification for ERCC1, BRCA1, RRM1 and RRM2, and β-actin was used as the reference gene. When comparing the threshold cycle with the standard curve (β-actin amount), the relative amount of cRNA of ERCC1, BRCA1, RRM1 and RRM2 was determined. Primers and probes of the ERCC1, BRCA1, RRM1 and RRM2 for polymerase chain reaction (PCR) amplification were designed using Sequenom Assay Design 3.1 software (Sequenom).The PCR reaction was started at 95℃ for 10 min to activate Taq polymerase, followed by 45 cycles of denaturation at 95℃ for 15 s, and annealing at 60℃ for 60s.

***Statistical analysis: ***Mean ± standard deviation (SD) was used to express the continuous variables, whereas frequencies and percentages were used to express the categorical variables. Multivariate logistic regression analysis was conducted to assess the association between ERCC1, BRCA1, RRM1 and RRM2 mRNA expression and response to chemotherapy, with adjusted odd ratios and their 95% confidence intervals (95%CI). The survival distribution was plotted by Kaplan-Meier methods and compared by log-rank test. Cox regression analysis was conducted to assess the association between ERCC1, BRCA1, RRM1 and RRM2 mRNA expression and overall survival, with hazard ratios (HR) and 95% confidence interval (95%CI). Statistical analyses were performed using the SPSS^®^ statistical package, version 11.0 (SPSS Inc., Chicago, IL, USA) for Windows. Two-tailed with a *P*-value <0.05 was considered as statistical significant.

**Table-I:** Characteristics of included patients

*Characteristics*	*Number* *N=208*	*Percentage (%)*
Age		
Median age(years)	64.1(25.3-86.1)	
≤60	91	43.7
>60	117	56.3
Gender		
Male	158	76.2
Female	50	23.8
Smoking status		
Never	139	66.8
Current or former	69	33.2
Stage		
IIIB	51	24.5
IV	157	75.5
Histopathology		
Adenocarcinoma	92	44.3
Squamous	98	47.3
Mixed/other NSCLC	17	8.4
Response to chemotherapy		
CR or PR	87	41.7
SD or PD	121	58.3

**Table-II:** ERCC1, BRCA1, RRM1 and RRM2 mRNA expression and response to chemotherapy

*Expression level*	*Total expression quantities*	*Responders*		*Non-responders*		*P value*	*OR(95%CI)*
		*N=87*	*%*	*N=121*	*%*		
High ERCC1	0.67±0.17	36	41.4	68	56.2		1.0(Ref.)
Low ERCC1		51	58.6	53	43.8	0.04	1.82(1.01-3.20)
High BRCA1	0.095±0.012	32	36.8	72	59.5		1.0(Ref.)
Low BRCA1		55	63.2	49	40.5	0.02	2.53(1.38-4.64)
High RRM1	0.24±0.17	40	46.0	64	52.9		1.0(Ref.)
Low RRM1		49	56.3	55	45.5	0.21	1.43(0.79-2.57)
High RRM2	2.45±0.32	42	48.3	62	51.2		1.0(Ref.)
Low RRM2		45	51.7	59	48.8	0.67	1.13(0.63-1.13)

**Table-III:** ERCC1, BRCA1, RRM1 and RRM2 mRNA expression and overall survival of advanced NSCLC

*Gene*	*Death*	*%*	*Alive*	*%*	*Overall Survival Median (month)*	*Log-rank*	*OS*	
	*N=113*		*N=95*				*HR(95%CI)*	*P value*
High ERCC1	66	58.4	38	40.0	16.05		1.0(Ref.)	
Low ERCC1	47	41.6	57	60.0	21.32	0.02	0.43(0.27-0.89)	0.008
High BRCA1	70	61.9	34	35.8	14.75		1.0(Ref.)	
Low BRCA1	43	38.1	61	64.2	22.61	0.004	0.37(0.22-0.66)	<0.001
High RRM1	62	54.9	42	44.2	17.25		1.0(Ref.)	
Low RRM1	51	45.1	53	55.8	19.65	0.17	0.65(0.39-1.23)	0.58
High RRM2	58	51.3	46	48.4	18.6		1.0(Ref.)	
Low RRM2	55	48.7	49	51.6	20.7	0.75	0.89(0.50-1.59)	0.68

**Fig.1:**
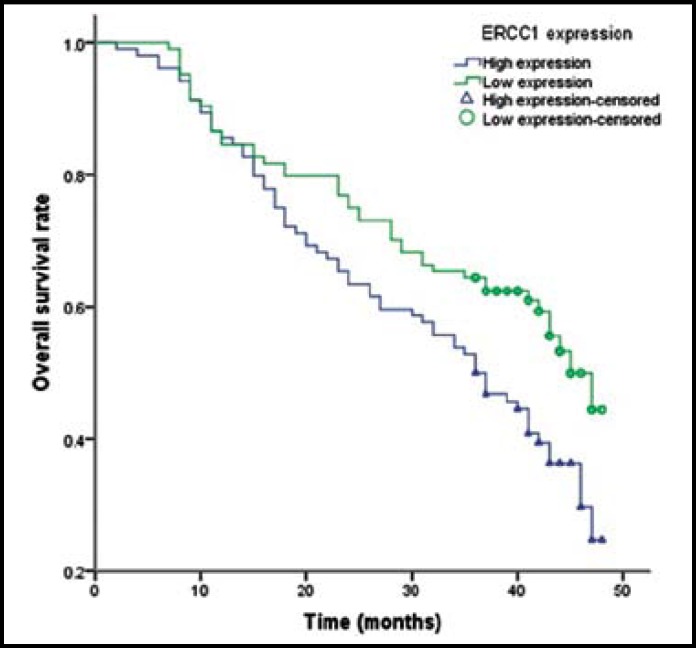
Kaplan-Meier curve for overall survival time of patients with different expression of ERCC1 mRNA

**Fig.2:**
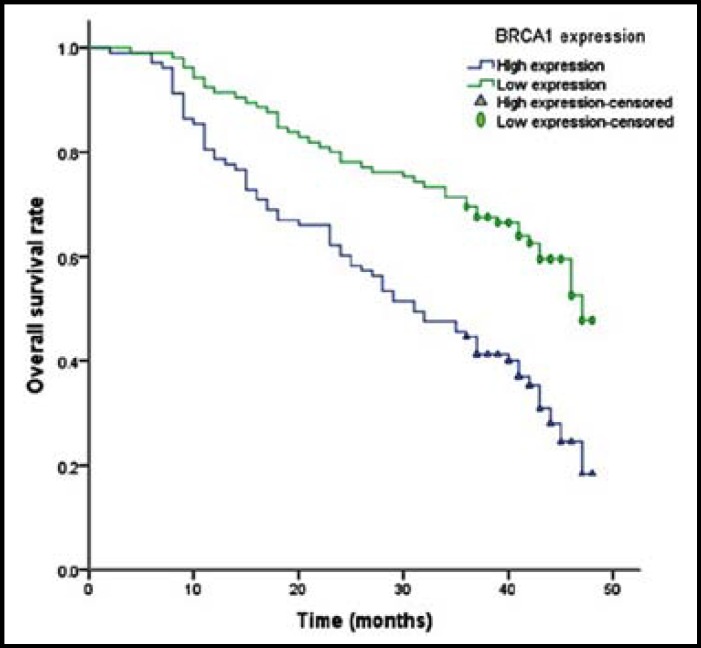
Kaplan-Meier curve for overall survival time of patients with different expression of BRCA1 mRNA

## RESULTS

***Patients: ***Characteristics of patients are summarized in Table-I. The median age of the enrolled patients was 64.1(25.3-86.1) years. Among these 208 patients, 117 (56.3%) were above the age of 60 years old, 158 (76.2%) were men, 69 (33.2%) were current or former smokers, 157 (75.5%) were at stage IV diseases and 190 (91.6%) were squamous and adenocarcinoma non-small cell lung cancer. After performing platinum-based doublets chemotherapy for these NSCLC patients, 87(41.7%) patients showed CR and PR to chemotherapy, and 121 (58.3%) achieved SD and PD to chemotherapy.

The standardized mRNA quantification amount of ERCC1, BRCA1, RRM1 and RRM2 was assessed by comparing the target amount of β-action amount. The expression of ERCC1, BRCA1, RRM1 and RRM2 was classified into high and low expression according to median expression level. The median expression levels of ERCC1, BRCA1, RRM1 and RRM2 were 0.67±0.17, 0.095±0.012, 0.24±0.17 and 2.45±0.32, respectively (Table-II). Our study found that the low ERCC1 and Low BRCA1 mRNA expression was more likely to be response to chemotherapy when compared with high expression, with the ORs (95% CI) of 1.82(1.01-3.20) and 2.53(1.38-4.64), respectively. However, we did not find the low level of RRM1 (OR=1.43, 95%CI=0.79-2.57) and RRM2 (OR=1.13, 95%CI=0.63-1.13) mRNA expression has a role on the response to platinum-based chemotherapy.

The median overall survival of enrolled patients was 17.9±7.5 months. We found that low expression of ERCC1 and BRCA1 mRNA had significantly longer overall survival time when compared with high expression of ERCC1 and BRCA1 mRNA (Table-III). Multivariate Cox regression analysis indicated that patients with low expression of ERCC1 and BRCA1 attained 0.43 (OR=0.43, 95%CI=0.27-0.89) and 0.37 (OR=0.37, 95%CI=0.22-0.66) fold risk of death from NSCLC (Fig.1 and Fig.2). However, no association was found between RMM1 and RRM2 mRNA expression and prognosis of NSCLC.

## DISCUSSION

Our study found an inverse correlation between ERCC1 and BRCA1 mRNA expression and response to platinum-based chemotherapy and clinical outcome of advanced NSCLC patients. Our study suggests expression of ERCC1 and BRCA1 mRNA could be helpful in predicting the clinical outcome of NSCLC and understand the pathogenesis of chemotherapy for NSCLC. Recently, several studies reported the association between ERCC1, BRCA1, RRM1 and RRM2 mRNA expression and clinical outcome of NSCLC.^3^^,^^8^^-^^13^ Zhang et al. reported that RRM1 and ERCC1 mRNA expression in tumor tissue could be predictive and prognostic biomarkers in advanced NSCLC receiving platinum-based chemotherapy.^3^ Boukovinas et al. reported that mRNA expression of BRCA1, RRM1 and RRM2 could be used to design individualized chemotherapy for NSCLC patients.^9^ Vassalou et al. reported that ERCC1 protein expression in tumor cell could influence the response rate to chemotherapy and clinical outcome of advanced NSCLC patients.^12^ However, the results are inconsistent. Therefore, we investigated the role of mRNA expression quantities of ERCC1, BRCA1, RRM1 and RRM2 in response to chemotherapy and clinical outcome of NSCLC patients.

ERCC1 is one of the key factors involved in nuclear excision repair and encodes the 5’endonuclease of the NER complex. It is reported that high expression of ERCC1 is corrected with resistance to cisplatin, which is involved in correcting the excision repair deficiency of the NER pathway.^15^ Previous studies reported that mRNA expression of ERCC1 was associated with response to chemotherapy and clinical outcome of head and neck cancer, colorectal cancer, gastric cancer and breast cancer.^16^^-^^19^ For patients with NSCLC, mRNA expression of ERCC1 might play an important role in the prognosis of NSCLCL patients treated with chemotherapy.^3^^,^^8^^,^^11^^-^^13^ One study conducted in China indicated that low expression of ERCC1 in peripheral blood or tumor tissue was associated with better response to chemotherapy longer median survival and longer progression-free survival.^3^ Another study conducted also conducted in China indicated that high ERCC1 protein expression was associated with clinical outcome of NSCLC patients treated with platinum-based chemotherapy.^10^ The previous two studies are in line with our findings. However, another study reported that ERCC1 expression was not prognostic of tumor recurrence and overall survival in patients with advanced NSCLC.^12^^,^^13^ The inconsistency of these findings could be explained by differences in ethnicities, number of included cases and study design. Therefore, further studies with different populations are greatly needed to confirm the finding of our study.

BRCA1 was considered as one important gene involved in regulating DNA damage responses and pivotal.^20^^,^^21^ Previous meta-analysis reported that the individuals with low or negative expression of BRCA1 were associated with longer OS and better objective response rate^20^ which is consistent with our study.

There are some limitations in our study. First, these advanced NSCLC patients were selected from one place, which may not better represent NSCLC patients in other populations. Second, the sample size of this study is relative small, which would reduce the statistical power to find the difference between groups. The relatively sample size may be the reason that no association was found between RRM1 and RRM2 mRNA expression and clinical outcome of NSCLC patients. Therefore, further large sample size and well designed studies are warranted.

In conclusion, our results indicate that mRNA expression of ERCC1 and BRCA1 could influence the efficacy of chemotherapy and clinical outcome of advanced NSCLC patients. Therefore, ERCC1 and BRCA1 mRNA expression could be important predictive markers for individualized platinum-based chemotherapy for NSCLC patients.
